# Theoretical Study on the Second Hyperpolarizailities of Oligomeric Systems Composed of Carbon and Silicon π-Structures

**DOI:** 10.3390/molecules21111540

**Published:** 2016-11-15

**Authors:** Hiroshi Matsui, Takanori Nagami, Shota Takamuku, Soichi Ito, Yasutaka Kitagawa, Masayoshi Nakano

**Affiliations:** Graduate School of Engineering Science, Osaka University, Toyonaka, Osaka 560-8531, Japan; hiroshi.matsui@cheng.es.osaka-u.ac.jp (H.M.); takanori.nagami@cheng.es.osaka-u.ac.jp (T.N.); shota.takamuku@cheng.es.osaka-u.ac.jp (S.T.); soichi@cheng.es.osaka-u.ac.jp (S.I.); kitagawa@cheng.es.osaka-u.ac.jp (Y.K.)

**Keywords:** diradical, second hyperpolarizability, silicon-silicon double bond, density functional theory

## Abstract

To explore the prospect of molecules involving silicon-silicon multiple bonds as nonlinear optical molecular systems, the relationship between the structure and the second hyperpolarizabilities γ of the oligomeric systems composed of carbon and silicon π-structures is investigated using the density functional theory method. It is found that these compounds indicate intramolecular charge transfer (ICT) from the silicon units to the carbon units together with nonzero diradical characters. The γ values of these compounds are shown to be 2–13 times as large as those of the carbon analogs. Although asymmetric carbon and silicon π-systems exhibit comparable enhancement to the corresponding symmetric systems, donor-π-donor structures exhibit remarkable enhancement of γ despite of their both-end short silicon π-chain moieties (donor units). Further analysis using the odd electron and γ densities clarifies that the intermediate diradical character also contributes to the enhancement of γ. These results predict that even short π-conjugated silicone moieties can cause remarkable enhancement of γ by introducing them into π-conjugated hydrocarbon structures.

## 1. Introduction

Multiple bonds are one of the essential elements for highly efficient functional molecules. Especially, multiple bonds between silicon (Si) atoms have been theoretically and experimentally investigated with great interest since Si containing compounds have several different chemical features from the carbon (C) analogs regardless of their belonging to the same 14 group. It is known that molecules with Si-Si multiple bonds take trans-bent structures [1] and indicate small HOMO-LUMO energy gap [2] as well as non-zero diradical character [3]. Since the first synthesis of disilene in 1981 [4], modern synthesis technique has enabled the synthesis of a variety of molecules with Si-Si multiple bonds, such as disilyne [2,5,6], tetrasilabuta-1,3-diene [7], π-conjugated systems [8], oligomers with long main chains [9], and so on. Such compounds are expected to have unique properties due to their stimuli-responsibilities of Si-Si multiple bonds. However, physicochemical properties deriving from Si-Si multiple bonds have not been revealed adequately. In general, π-electrons in multiple bonds are known to be well related to absorption-emission spectra and other optical properties of the molecules. Among them, nonlinear optical (NLO) properties have attracted much interest due to their future potential for applications in photonics and optoelectronics, e.g., modern harmonic imaging [10], huge capacity optical-data storage [11,12], and ultrafast optical switching [13]. For enhancement or control of NLO properties, several molecular design principles have been proposed, e.g., extension of π-conjugation [14,15,16], donor(D)/acceptor(A) substitution [14,15,16,17], and controlling charge states [16,18]. Although these traditional design principles have targeted only closed-shell systems, our previous studies have shed light on open-shell systems and have revealed the relationship between diradical character (*y*) and NLO properties: the system with intermediate *y* (0 < *y* < 1) indicates larger enhancement of second hyperpolarizability (γ, the microscopic origin of the third-order NLO phenomena) than closed-shell (*y* = 0) and pure-open shell (*y* = 1) systems of similar size [19,20,21,22]. On the basis of this design principle, a variety of molecular systems with intermediate *y* have been theoretically and experimentally reported as highly active NLO molecular systems [20,21,22,23]. Recently, we have reported that poly(disilene-1,2-diyl), the silicon analog of polyacetylene, exhibits much greater enhancement of γ than polyacetylene and polysilane because of its intermediate *y* values [24]. However, synthesis of poly(disilene-1,2-diyl) with long main chain is still a challenging topic in modern chemistry due to its instability, though stable π-conjugated systems composed of C and Si π-structures have been synthesized as mentioned above. Therefore, introducing π-conjugated Si moiety into C π-structures may be a prospective way to enhance NLO properties due to its more realizability than poly(disilene-1,2-diyl). In this study, therefore, we investigate the effects of introducing Si–Si double bonds into C oligomeric π-structures on the γ values from the viewpoint of open-shell singlet nature and intramolecular charge transfer (ICT) nature. Such systems with long main chains have already been synthesized [9,25], and are expected to have ICT nature, which is known as one of the important factors of enhancement of NLO properties and as a molecular design guideline for highly active NLO systems such as D-π-D, A-π-A, and D-π-A structures [14,17]. The present results will contribute to building a novel design guideline for stable efficient NLO systems as well as to designing a new type of realistic Si based NLO molecular systems.

## 2. Results and Discussion

**Figure 1:**
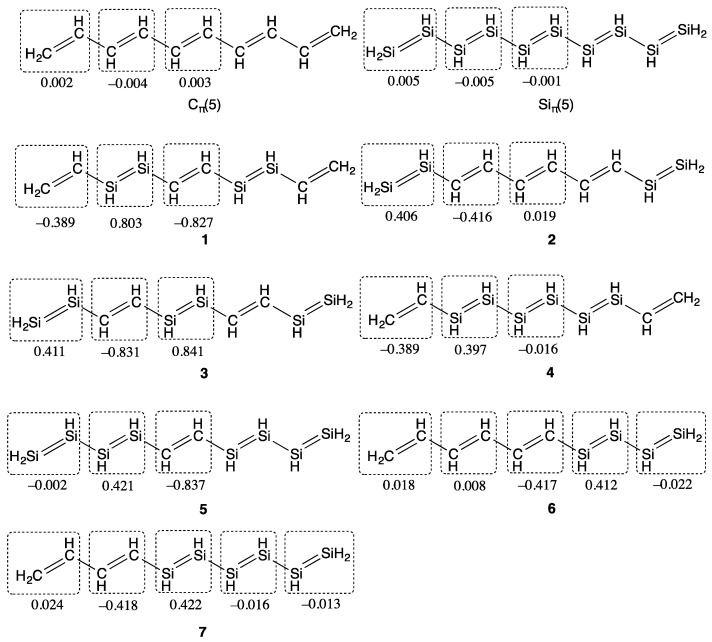
π-Conjugated molecules examined in this study. Natural charge of each unit is shown. Natural charges are calculated using the LC-(U)BLYP(μ = 0.33)/aug-cc-pVDZ method.

Figure 1 shows the compounds examined in this study. These compounds are composed of both ethylene units (C units) and disilene units (Si units). The compounds **1**–**5** are symmetric systems, while **6** and **7** are asymmetric systems. For comparison, the results of C_π_(5) and Si_π_(5) investigated in our previous study [24] are also shown.

Figure 1 also shows the charge distribution of each unit in these compounds. Although C_π_(5) and Si_π_(5) hardly indicate ICT, the combined systems **1**–**7** indicate ICT from Si units to C units. The natural charge of each Si (C) unit is shown to almost depend on the number of adjacent C (Si) units. In **1** and **3**, for example, the central C (Si) unit is found to be approximately negatively (positively) charged twice as much as the terminal Si (C) units. It is notable that ICT in **1**–**7** occurs not in the whole region of the molecule but between the adjacent units. In the compounds **2**, **4**, and **5**, it turns out that Si units adjacent to C units donate charge to the adjacent C units and are positively charged with similar amplitudes (0.406, 0.397, and 0.421, respectively), while that Si units adjacent to Si units in **4** (central Si unit) and **5** (terminal Si units) are hardly charged. The same discussion can be deduced for **6** and **7**. It is found that only Si units adjacent to C units are positively charged (0.412 in **6** and 0.422 in **7**, respectively), while that the other Si units are hardly charged. There is shown to be little difference between their sum of charge in Si units (0.390 in **6** and 0.393 in **7**) and between the longitudinal component of dipole moment amplitudes (2.93 in **6** and 2.81D in **7**). These results show that ICT occurs only between the Si unit and the adjacent C unit.

**Figure 2:**
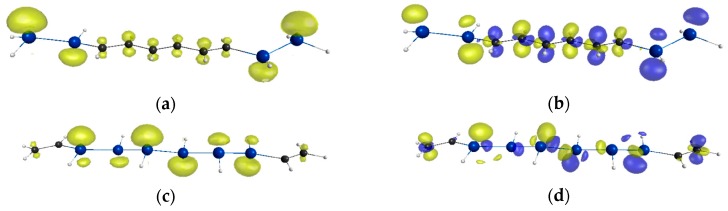
Comparison between the odd electron density distribution and γ density distribution of **2** and **4**: (**a**) Odd electron density distribution of **2** with the contour value of 0.001 a.u.; (**b**) Positive (yellow) and negative (blue) γ density distributions of **2** with the contour value of ±3000 a.u.; (**c**) Odd electron density distribution of **4** with the contour value of 0.0025 a.u.; (**d**) Positive (yellow) and negative (blue) γ density distributions of **4** with the contour value of ±3000 a.u.

Table 1 lists the *y* and γ values of each compound. The combined systems **1**–**7** indicate nonzero *y* like Si_π_(5) though they are smaller than that of Si_π_(5). The compound with longer Si chain and more Si units tends to exhibit larger *y*. As seen from Figure 2 (see also the Supplementary Materials except for **2** and **4**), the odd electron density of the combined systems are distributed not only on Si units but also on C units, which means that the radical delocalizes over the whole main chain. This is also supported by the *y* values. The compounds **1**–**3** indicate larger *y* than Si_π_(1) (*y* = 0.0768 [24]), and **5** also indicates larger *y* than Si_π_(2) (*y* = 0.209 [24]). These results show that separated Si units interact with each other through the C units.

The γ values of the combined systems are shown to lie in between those of C_π_(5) (γ = 2.27 × 10^5^ a.u.) and Si_π_(5) (γ = 48.5 × 10^5^ a.u.). These γ values are found to depend on their molecular structures though γ is shown to basically increase with the number of Si units as well as *y*. In addition, similar spatial distributions of the odd electron and γ densities (see Figure 2 for **2** and **4** as the representatives, see also the Supplementary Materials for the other compounds) indicate that the primary contribution to γ comes from the radical electrons.

The comparison of the γ values between isomers **1** and **2** gives noteworthy information. The γ value of compound **2** is shown to be twice as large as that of **1**, which shows alternate change of sign of charge for every double bond unit and this feature tends to cancel the ICT effect with each other in the molecule. The compound **2** indicates ICT nature with a D-π-D structure. Moreover, it is found that the γ value of **2** is comparable to that of **4**, though **4** has a longer Si chain length and a larger *y* than **2**. These results clarify the γ enhancement effect of D-π-D structure. These explanations rationally accord with the result of **5**, which also has a D-π-D structure and indicates a larger *y* than **2**, and which exhibits the largest γ of these compounds. These D-π-D systems **2** and **5** exhibit more than 5 times and 13 times enhanced γ as compared to C_π_(5). Especially, the γ value of **5** is comparable to that of Si_π_(5). These results imply that even short π-conjugated Si-chain can cause enhancement of γ by introducing it into C π-structures and that the introduction of Si moiety into the both-end region of C π-structures is better to enhance the γ than that into the central region of C π-structures since the former constructs a D-π-D structure. This enhancement can be explained as follows. Although radical electrons delocalize in the whole region of the main chain, their distributions on Si units are more significant than those on C units (Figure 2a,c). Therefore, the odd electron density of a D-π-D structure such as **2** tends to be primarily distributed in the both-end region, while that of an A-π-A structure such as **4** tends to be primarily distributed in the middle region. As mentioned above, their γ density distributions show similar tendencies to their odd electron densities, and the one end region has an opposite sign to the other end region. Since the large distance between the positive and negative γ densities with larger amplitudes gives larger contribution to γ, the introduction Si units into the both-end region is found to be superior to that into the central region. Consequently, D-π-D structures constructed from the introduction of Si units into the both-end region tend to exhibit larger enhancement of γ than A-π-A structures constructed from the introduction into the central region though ICT occurs in both systems. As a result, such a short π-conjugated Si moiety is expected as a building block of highly efficient π-conjugated NLO systems.

On the other hand, asymmetric systems **6** and **7** indicate merely comparable enhancement of γ to symmetric systems though asymmetricity or large ICT is found to be able to cause further enhancement of hyperpolarizabilities in general [17,26]. It turns out that the compound **6** has a slightly smaller γ than **2** despite its longer Si chain, and that **7** also exhibits a comparable γ to **3**. These results can be attributed to their weak asymmetricity. As mentioned before, ICT in **6** and **7** almost occurs not over the whole region of the molecule but between the adjacent Si- and C-units. Thus, their dipole moment amplitudes are not shown to be so large (2.93 and 2.81D, respectively), so that they are not shown to cause remarkable enhancement of γ.

**Figure 3:**
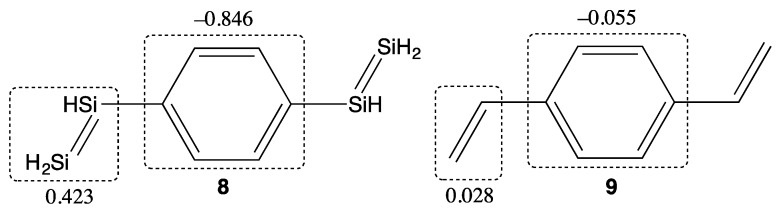
Molecular structure and natural charge distributions of the compounds **8** and **9**. Natural charges are calculated using the LC-UBLYP(μ = 0.33)/aug-cc-pVDZ method.

**Figure 4:**
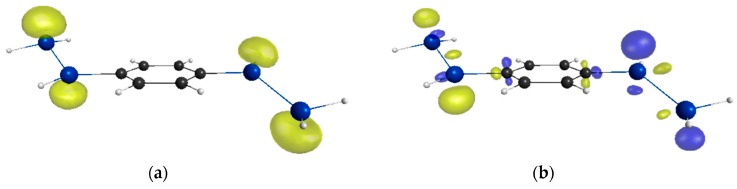
Comparison between the odd electron density distribution and the γ density distribution of **8**: (**a**) Odd electron density distribution of **8** with the contour value of 0.0002 a.u.; (**b**) Positive (yellow) and negative (blue) γ density distributions of **8** with the contour value of ±1500 a.u.

Finally, we investigate a realistic system involving both C and Si π-systems, the compound **8,** whose main skeleton has been synthesized [25]. For comparison with the carbon analog, the compound **9** is investigated. As seen from Table 2, compound **8** indicates nonzero *y* (*y* = 0.160) and the γ is shown to be about five times as large as that of **9**. This result is attributed to the D-π-D structure of **8**. As seen from Figure 3, ICT occurs from the central benzene ring to the terminal Si unit, while such ICT does not occur in **9**. In addition, the similarity between the odd electron and γ density distributions of **8** indicates the contribution of the diradicals with intermediate diraidcal character to the enhancement of γ (see Figure 4).

## 3. Theories and Calculation Methods

Geometrical optimizations were conducted at RB3LYP/cc-pVDZ level of theory without any symmetry constraints. The diradical character *y* [27,28] is defined as the occupation number of the lowest unoccupied natural orbital (LUNO) by quantum chemical calculation:

*y* = *n*_LUNO_ (1)

Broken symmetry methods like the spin-unrestricted (U) HF method generally suffer from spin contamination in electronic structure calculation for open-shell systems. In such cases, an approximate spin-projection scheme [29] is known to improve the results. The *y* values by the spin-projected (P) UHF method are given by

(2) $$y=1-\frac{2T}{1+T^{2}}$$

where *T* denotes the overlap between the highest occupied corresponding orbitals for the α and β spins [29]. In this study, the *y* values are calculated at PUHF/cc-pVDZ level of theory. The finite field approach [30] was adopted to obtain the static longitudinal second hyperpolarizability γ with the long-range corrected (LC)-UBLYP(μ = 0.33)/aug-cc-pVDZ method because the LC-UBLYP(μ = 0.33) method is known to semiquantitatively reproduce the result by the highly correlated wavefunction method, UCCSD(T), for open-shell systems [31] and because the diffuse functions are necessary for accurate evaluation of high-order optical response properties [32]. The longitudinal axis is defined to be along the line connecting the two terminal 14 group elements in the main chain. The natural charges and dipole moments were calculated by the LC-(U)BLYP(μ = 0.33)/aug-cc-pVDZ method. To clarify the spatial distribution of radical (unpaired) electrons, odd electron density analyses [33,34] were performed using the LC-UBLYP(μ = 0.33)/aug-cc-pVDZ method. The odd electron density *D*_odd_(***r***) is defined by the *m*th NO *φ_m_* and its occupation number *n_m_* as

(3) $$D_{\mathrm{odd}}\left(r\right)=\sum_{m}\min\left(n_{m},2-n_{m}\right)\phi_{m}^{*}\left(r\right)\phi_{m}\left(r\right)$$

Here, min(*n_m_*, 2 − *n_m_*) can be regarded as the probability for the electron of being unpaired in *φ_m_*. To clarify the spatial contribution of electrons to γ, the γ density analyses [35] are also performed using the LC-UBLYP(μ = 0.33)/aug-cc-pVDZ method. The γ density *ρ_iii_*(***r***) is defined as

(4) $$\rho_{iii}\left(r\right)={\frac{\partial^{3}\rho }{\partial F_{i}^{3}}|}_{F=0}$$

where *ρ* and *F_i_* denote the electron density and the longitudinal component of the external static electric field, respectively. The γ value can be expressed by *ρ_iii_*(***r***) as

(5) $$\gamma =-\frac{1}{3!}\int r_{i}\rho_{iii}\left(r\right)dr$$

where *r_i_* denotes the longitudinal component of the coordinate of an electron. All the quantum chemical calculations were performed by the Gaussian 09 program package [36].

## 4. Conclusions

Using the density functional theory methods, the effects of introducing Si-Si double bonds into C π-structures on γ are investigated. In such compounds, ICT from Si unit to C unit is found to occur. These compounds indicate relatively small diradical character as compared to Si_π_(5) and exhibit enhancement of γ as compared to C_π_(5), e.g., one of their γ values are found to be comparable to that of Si_π_(5), which shows about 20 times enhancement as compared to that of C_π_(5). The comparison between the odd electron and γ densities shows the contribution of diradicals with intermediate diradical character to the enhancement of γ. It is also found from the comparison for the systems with the same number of Si units that D-π-D structures, **2** and **5**, are more effective to the enhancement of γ than the corresponding asymmetric systems, **6** and **7**, respectively, which show comparable enhancement of γ to the other symmetric systems with the same number of Si units, **1** and (**3**, **4**), resepectively. In addition, the introduction of relevant-length Si units into the both-end regions of C π-structures (**5**) are found to be more effective to enhancing γ than that into the central region of C π-structures (**4**) since the former constructs a D-π-D structure with abundant distribution of radical electrons in the both-end region. In realistic compounds, for example, the D-π-D system composed of benzene and Si=Si units (**8**) exhibits about five times greater enhancement of γ than its carbon analog (**9**). Considering the previous studies on synthesis of the systems composed of both Si and C π-structures [8,9,25], such π-conjugated Si-chain substituted hydrocarbon systems are expected to be more stable than the corresponding π-conjugated Si moiety of similar-size and to be realizable as a novel type of highly efficient open-shell NLO molecular systems.

## Figures and Tables

**Table 1:** *y* (−) and γ (10^5^ a.u.) values of the compounds shown in Figure 1.

Compounds	*y* ^2^	γ/10^5^ a.u. ^3^
C_π_(5)	0.000 ^1^	2.27 ^1^
Si_π_(5)	0.495 ^1^	48.5 ^1^
**1**	0.182	5.90
**2**	0.254	12.8
**3**	0.235	15.0
**4**	0.340	12.1
**5**	0.344	31.3
**6**	0.287	9.58
**7**	0.360	15.6

^1^ Reference [24]; ^2^ Calculated using the PUHF/cc-pVDZ method; ^3^ Calculated using the LC-UBLYP(μ = 0.33)/aug-cc-pVDZ method.

**Table 2:** *y* and γ values of the compound **8** and **9**.

Compound	*y* ^1^	γ/10^5^ a.u. ^2^
**8**	0.160	1.81
**9**	0.000	0.366

^1^ Calculated using the PUHF/cc-pVDZ method; ^2^ Calculated using the LC-UBLYP(μ = 0.33)/aug-cc-pVDZ method.

## Supplementary Materials

Supplementary materials can be accessed at: http://www.mdpi.com/1420-3049/21/11/1540/s1, Figures S1–S5: Comparison between odd electron and γ density distributions of the compounds **1**, **3**, and **5**–**7**, Tables S1–S9: optimized structures of the compounds **1**–**9**.
