# Supplementary material for: Theoretical Study on the Second Hyperpolarizailities of Oligomeric Systems Composed of Carbon and Silicon π-Structures
Source: Molecules. 2016 Nov 15;21(11):1540. doi: 10.3390/molecules21111540 (PMC6274231; doi:10.3390/molecules21111540)
Supplement: Supplementary file 1 [file molecules-21-01540-s001.pdf]

Supplementary Materials: Theoretical Study on the Second Hyperpolarizailities of Oligomeric Systems Composed of Carbon and Silicon π-Structures

Hiroshi Matsui, Takanori Nagami, Shota Takamuku, Soichi Ito, Yasutaka Kitagawa and Masayoshi Nakano

1. Comparison between Odd Electron and γ Density Distributions of the Compounds

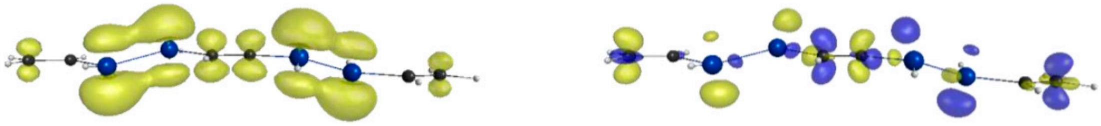

(**a**) (**b**)

Figure S1. (**a**) Odd electron density distribution of **1** with the contour value of 0.00025 a.u.; (**b**) Positive (yellow) and negative (blue) γ density distributions of **1** with the contour value of ±1500 a.u.

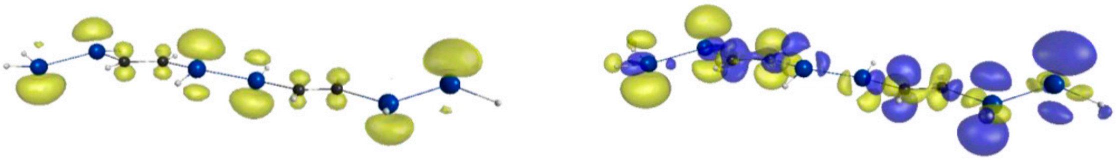

(**a**) (**b**)

Figure S2. (**a**) Odd electron density distribution of **3** with the contour value of 0.001 a.u.; (**b**) Positive (yellow) and negative (blue) γ density distributions of **3** with the contour value of ±1500 a.u.

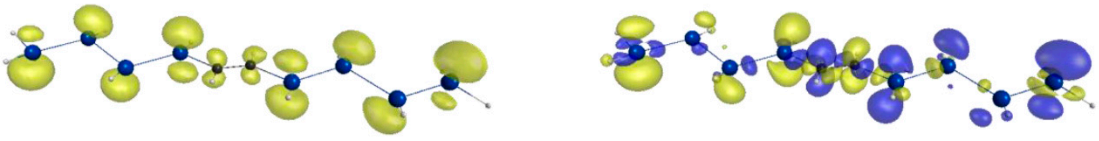

(**a**) (**b**)

Figure S3. (**a**) Odd electron density distribution of **5** with the contour value of 0.001 a.u.; (**b**) Positive (yellow) and negative (blue) γ density distributions of **5** with the contour value of ±3000 a.u.

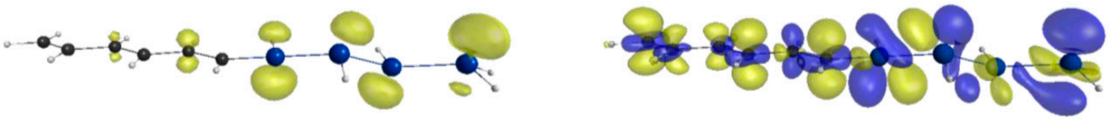

(**a**) (**b**)

Figure S4. (**a**) Odd electron density distribution of **6** with the contour value of 0.001 a.u.; (**b**) Positive (yellow) and negative (blue) γ density distributions of **6** with the contour value of ±1500 a.u.

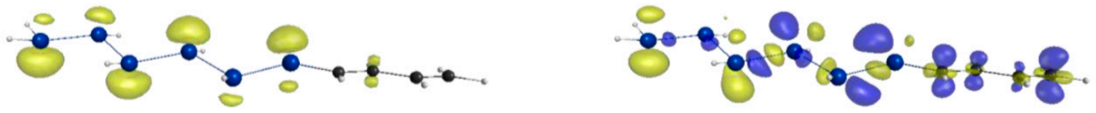

(**a**) (**b**)

Figure S5. (**a**) Odd electron density distribution of **7** with the contour value of 0.0025 a.u.; (**b**) Positive (yellow) and negative (blue) γ density distributions of **7** with the contour value of ±1500 a.u.

2. Optimized Structures

Table S1. Optimized Structure of **1** Calculated Using the RB3LYP/cc-pVDZ Method.

Atom	*x*/Å	*y*/Å	*z*/Å
C	0.5120750521	0.0000000085	−0.6139153659
C	0.1721311894	0.4823816029	0.6139214986
Si	0.7544162094	−0.2304086101	2.2199318262
Si	0.0366732701	0.7880928791	−2.2199604639
Si	−0.2357688251	0.2264550640	4.1113803530
Si	0.1340099880	−0.2980996340	−4.1114006501
C	0.3390934650	−0.4885564352	5.7273405111
C	−0.0000000013	0.0000000019	6.9348608600
C	−0.3459963904	0.4837036468	−5.7273653270
C	0.0000000013	−0.0000000019	−6.9348608600
H	1.1490537473	−0.8933556322	−0.6713404293
H	−0.4549551753	1.3827086281	0.6713396198
H	1.4607047848	−1.5381304576	2.0648986291
H	−0.9573255441	1.8930502257	−2.0649681675
H	−0.9410163754	1.5348349353	4.2654868072
H	1.1290006692	−1.4022753991	−4.2654550513
H	0.9585718080	−1.3934597177	5.6844479204
H	−0.6204260685	0.8958021632	7.0444452733
H	0.3324132782	−0.4760214432	7.8632099569
H	−0.9898082282	1.3714565959	−5.6844749211
H	0.6349223205	−0.8855868103	−7.0444371938
H	−0.3364541561	0.4731641772	−7.8632151701

Table S2. Optimized Structure of **2** Calculated Using the RB3LYP/cc-pVDZ Method.

Atom	*x*/Å	*y*/Å	*z*/Å
C	0.5686886126	0.0000000022	0.6182478880
C	0.2778424476	0.4964656620	−0.6184942176
C	0.6502993008	−0.1458323049	−1.8513465126
C	0.1900310945	0.6386087762	1.8511486892
C	0.3599031409	0.3359233003	−3.0944594945
C	0.4685503695	0.1498021660	3.0942896294
Si	0.0576476364	1.0184575195	4.6682690739
Si	0.9172338668	−0.4467775748	−4.6682007597
Si	−0.0000000026	0.0000000045	6.6085675470
Si	0.0000000026	−0.0000000045	−6.6085675470
H	1.1225573081	−0.9435144055	0.6931555265
H	−0.2746924243	1.4407360564	−0.6935167457
H	1.2020257638	−1.0899604422	−1.7569944836
H	−0.3642618573	1.5812283797	1.7567917691
H	−0.1878618777	1.2852255062	−3.1589612295
H	1.0293061465	−0.7918813932	3.1588246501
H	−0.8395759651	2.1926309155	4.4350943515
H	1.5031122355	−1.8033784005	−4.4349094660

H	1.0158631920	−1.0726864079	6.8402843997
H	−0.1724926360	0.8764275845	7.8067831064
H	−0.4392465037	1.4105673223	−6.8404334954
H	0.6810039267	−0.5785087786	−7.8065828887

Table S3. Optimized Structure of **3** Calculated Using the RB3LYP/cc-pVDZ Method.

Atom	*x*/Å	*y*/Å	*z*/Å
Si	0.5385697648	−0.0000000101	0.9492063512
Si	−0.5382399938	0.0000930897	−0.9492644312
C	0.2680679693	−0.4893606313	−2.5394025986
C	−0.2677546797	0.4894182726	2.5392280991
C	−0.2531209575	−0.2523328986	−3.7759794166
C	0.2533454890	0.2523364376	3.7758454872
Si	−0.4979462168	0.8635713971	5.3555739915
Si	0.4980445797	−0.8634940050	−5.3556799170
Si	−0.0000000053	0.0000000037	7.3057042985
Si	0.0000000053	−0.0000000037	−7.3057042985
H	1.7451832632	−0.8619362828	1.1175061140
H	−1.7448621267	0.8620028752	−1.1176850074
H	1.2152506626	−1.0408265722	−2.4631875352
H	−1.2148401573	1.0410475578	2.4629950415
H	−1.2057519008	0.2894797391	−3.8524948097
H	1.2058663663	−0.2896644873	3.8524382522
H	−1.8637301383	1.4318100000	5.1344012109
H	1.8637656918	−1.4318733007	−5.1345850178
H	1.4103118763	−0.4468651245	7.5198240503
H	−0.5640881256	0.6835803847	8.5085064426
H	−1.4102997655	0.4469305997	−7.5197095499
H	0.5640235670	−0.6835662899	−8.5085306609

Table S4. Optimized Structure of **4** Calculated Using the RB3LYP/cc-pVDZ Method.

Atom	*x*/Å	*y*/Å	*z*/Å
Si	−0.5024757768	0.0000782107	−0.9925713798
Si	0.5024405400	0.0000000082	0.9925738362
Si	−0.7543289372	0.1724507888	2.9091302826
Si	0.7543094770	−0.1723685680	−2.9091184571
Si	0.2897971349	−0.0019431806	4.8373682819
C	−0.4874423673	0.4122189984	6.4734765613
Si	−0.2897900537	0.0020276240	−4.8373702706
C	−0.0000000047	0.0000000021	7.6593004784
C	0.4874798795	−0.4121293968	−6.4734656213
C	0.0000000047	−0.0000000021	−7.6593004784
H	−1.5737499117	1.0414083353	−1.1375713991
H	1.5737167291	−1.0413277696	1.1375739116
H	−1.7932609604	1.2521620051	2.8767141607
H	1.7932408082	−1.2520804660	−2.8766913715
H	1.3991157743	−0.9936000256	4.9854977811
H	−1.3690274603	1.0653638377	6.4664712497
H	−1.3991099831	0.9936805108	−4.9855164151
H	0.8799717133	−0.6484403265	7.7293611725
H	−0.4629378294	0.2937147429	8.6072815896
H	1.3689458077	−1.0654378268	−6.4664566737
H	−0.8798131879	0.6486596736	−7.7293689099
H	0.4628712833	−0.2938437794	−8.6072765928

Table S5. Optimized Structure of **5** Calculated Using the RB3LYP/cc-pVDZ Method.

Atom	*x*/Å	*y*/Å	*z*/Å
C	0.3070839067	0.0000000019	−0.6096842110
C	−0.3070729693	−0.0000109909	0.6096781599
Si	0.5750368763	−0.2129491379	2.2191843213
Si	−0.5750226248	0.2129477606	−2.2191843200
Si	−0.3396667509	0.2214551126	4.1715623031
Si	0.7672212211	−0.4870174766	6.0553183761
Si	0.3396750269	−0.2214587840	−4.1715633175
Si	−0.0000000011	−0.0000000019	8.0622577070
Si	−0.7672123650	0.4870227147	−6.0553163433
Si	0.0000000011	0.0000000019	−8.0622577070
H	1.4020926776	−0.0782103590	−0.6491880136
H	−1.4020868522	0.0781965715	0.6491809446
H	2.0607597256	−0.1670515967	2.0686021219
H	−2.0607453986	0.1670582655	−2.0685990784
H	−1.8281048696	0.0654292895	4.2121810412
H	2.2542880843	−0.3855287494	5.8979363023
H	1.8281142918	−0.0654428845	−4.2121841069
H	−1.4709936275	−0.1428129625	8.2915697186
H	0.7817501475	−0.4810037343	9.2437999247
H	−2.2542786487	0.3855428917	−5.8979302224
H	1.4709935531	0.1428049269	−8.2915747608
H	−0.7817510807	0.4810100535	−9.2437968921

Table S6. Optimized Structure of **6** Calculated Using the RB3LYP/cc-pVDZ Method.

Atom	*x*/Å	*y*/Å	*z*/Å
C	0.1210505073	0.0000000019	−1.8663425928
C	−0.2908608471	0.4625720910	−0.6504605902
Si	0.3873227591	−0.1215507292	0.9569058901
C	−0.3585003245	0.4849374451	−3.1361335700
Si	−0.5654817907	0.2137374795	2.9158011732
Si	0.7686127660	−0.1904992035	4.7456262217
C	0.0590674624	0.0025793921	−4.3369763595
Si	−0.0000000057	0.0000000044	6.8058774315
C	−0.4166502301	0.4797209058	−5.6182993043
C	0.0000000057	−0.0000000044	−6.8058774315
H	0.8690785157	−0.8023348073	−1.9084900435
H	−1.0362117714	1.2681317693	−0.6332626400
H	1.3049583046	−1.2898342215	0.7834075801
H	−1.1045773208	1.2883402796	−3.1154461546
H	−1.3591754347	1.4830447688	2.9898484688
H	1.6532378414	−1.3807515647	4.5169917527
H	0.8055327767	−0.8010393370	−4.3531225460
H	−0.8114980468	1.2195334701	7.1154245307
H	0.9645394470	−0.2872826687	7.9152011110
H	−1.1627215422	1.2826600197	−5.5960187159
H	0.7429925098	−0.8007758471	−6.8663659535
H	−0.3882182266	0.3924400887	−7.7476422539

Table S7. Optimized Structure of 7 Calculated Using the RB3LYP/cc-pVDZ Method.

Atom	*x*/Å	*y*/Å	*z*/Å
Si	1.0156358839	−0.0000000024	0.2906898435
Si	−0.0887489878	−0.1010751600	2.1946833337
C	0.6256030272	0.3194655431	3.8408453429
Si	−0.2459496122	−0.1376824355	−1.6191667795

C	0.0528815979	−0.0190128195	5.0275248317
C	0.5772346530	0.3482066143	6.3302888946
Si	0.6626412150	−0.1176429300	−3.6457549413
C	0.0000000001	−0.0000000005	7.4956223876
Si	−0.7783110618	−0.1270792495	−5.4410861441
Si	−0.0000000001	0.0000000005	−7.4956223876
H	2.0967125096	1.0362917132	0.2631025592
H	−1.2643941999	−1.0175511186	2.3084281911
H	1.5443287832	0.9197263932	3.8645901542
H	−1.3907242141	−1.0922882588	−1.4525376650
H	−0.8697152215	−0.6138473339	5.0317309215
H	1.4985318979	0.9420954812	6.3395008169
H	1.7739796697	0.8767982275	−3.8085183222
H	−0.9198096633	−0.5920229406	7.5200717506
H	0.4284176697	0.2956217907	8.4553881781
H	−1.9181306496	−1.0845182412	−5.2628796714
H	1.0950375154	0.9787763628	−7.7750314204
H	−0.9658608157	−0.0769678501	−8.6354552750

Table S8. Optimized Structure of 8 Calculated Using the RB3LYP/cc-pVDZ Method.

Atom	*x*/Å	*y*/Å	*z*/Å
C	0.5120950115	0.0000000105	−1.3296054125
C	1.2360121027	0.5885125169	−0.2694393219
C	0.7319820955	0.5942864141	1.0292521429
C	−0.5120939997	0.0002277549	1.3296349361
C	−1.2360136036	−0.5882772054	0.2694674773
C	−0.7319793997	−0.5940570828	−1.0292234635
H	2.2035574898	1.0579244923	−0.4659412066
H	1.3131217683	1.0734770697	1.8218997368
H	−2.2035650519	−1.0576797601	0.4659665487
H	−1.3131204490	−1.0732461165	−1.8218722516
Si	−1.2205319477	0.0292521829	3.0640572393
Si	1.2205730405	−0.0290633255	−3.0640395624
Si	0.0000000062	0.0000000021	4.8859614126
Si	−0.0000000062	−0.0000000021	−4.8859614126
H	−2.5387757347	−0.6778491762	3.0965695815
H	2.5387669069	0.6781424993	−3.0965771645
H	1.2710763924	0.7898733708	4.8699660485
H	−0.7584707364	0.2077805021	6.1579968542
H	−1.2708588954	−0.7902490681	−4.8698165186
H	0.7585115101	−0.2078562445	−6.1579691743

Table S9. Optimized Structure of 9 Calculated Using the RB3LYP/cc-pVDZ Method.

Atom	*x*/Å	*y*/Å	*z*/Å
C	0.0000000000	0.0000000000	−3.8820204700
C	0.7816861300	0.0000000000	−2.7911381700
C	0.3561403800	0.0000000000	−1.3827735200
C	1.3357679800	0.0000000000	−0.3690285700
C	0.9925612200	0.0000000000	0.9790126100
C	−0.3561403800	0.0000000000	1.3827735200
C	−1.3357679800	0.0000000000	0.3690285700
C	−0.9925612200	0.0000000000	−0.9790126100
C	−0.7816861300	0.0000000000	2.7911381700
C	0.0000000000	0.0000000000	3.8820204700
H	−1.0913630700	0.0000000000	−3.8257088700
H	0.4384802900	0.0000000000	−4.8819179500
H	1.8681963700	0.0000000000	−2.9363241200

H	2.3917110000	0.0000000000	−0.6530240800
H	1.7865030800	0.0000000000	1.7283012900
H	−2.3917110000	0.0000000000	0.6530240800
H	−1.7865030800	0.0000000000	−1.7283012900
H	−1.8681963700	0.0000000000	2.9363241200
H	1.0913630700	0.0000000000	3.8257088700
H	−0.4384802900	0.0000000000	4.8819179500
